# Olmesartan/Amlodipine/Hydrochlorothiazide in Obese Participants With Hypertension: A TRINITY Subanalysis

**DOI:** 10.1111/jch.12133

**Published:** 2013-06-04

**Authors:** Eli M Roth, Suzanne Oparil, Michael Melino, James Lee, Victor Fernandez, Reinilde Heyrman

**Affiliations:** 1 Sterling Research GroupCincinnati, OH; 2 The University of Alabama at BirminghamBirmingham, AL; 3 Daiichi Sankyo IncParsippany, NJ; 4 Formerly of Daiichi Sankyo IncParsippany, NJ

## Abstract

The objective of this prespecified TRINITY study subgroup analysis was to assess the efficacy and safety of triple-combination treatment with olmesartan medoxomil (OM) 40 mg, amlodipine besylate (AML) 10 mg, and hydrochlorothiazide (HCTZ) 25 mg vs the component dual-combination treatments in obese (body mass index [BMI] ≥30 kg/m^2^) and nonobese (BMI <30 kg/m^2^) hypertensive participants. The double-blind treatment period primary end point was the least-squares (LS) mean reduction in seated diastolic BP (SeDBP) at week 12 (end of the double-blind period). Of the 2492 randomized participants, 1555 (62.4%) had BMI ≥30 kg/m^2^. Irrespective of BMI, triple-combination treatment resulted in greater LS mean reductions in seated BP (SeBP) (≥30 kg/m^2^, 6.7–10.5/4.5–7.3 mm Hg; <30 kg/m^2^, 5.1–8.6/2.5–6.0 mm Hg [*P*<.005] vs dual-combination treatments for both subgroups) at week 12. Furthermore, triple-combination treatment enabled a greater proportion of participants to reach BP goal vs the dual-combination treatments (≥30 kg/m^2^, 62% vs 31%–46% [*P*<.0001]; <30 kg/m^2^, 69% vs 41%–55% [*P*<.005]) at week 12. SeBP reduction and goal attainment (≥30 kg/m^2^, 63%; <30 kg/m^2^, 67%) was maintained through week 52/early termination. Triple-combination treatment was well tolerated in both BMI subgroups.

Obesity (body mass index [BMI] ≥30 kg/m^2^) and hypertension are common and frequently coexistent disorders.^1^,^2^ During the past 20 years, the prevalence of obesity in the United States has increased substantially, and approximately one third of US adults are currently obese.^3^–^4^ Similarly, data from the National Health and Nutrition Examination Survey (NHANES) indicate that 33.0% of US adults (77.9 million individuals) have hypertension, and this prevalence is projected to increase to 37.3%, yielding an additional 27 million individuals with hypertension by the year 2030.^3^–^5^ Numerous evaluations have demonstrated a direct correlation between BMI and hypertension.^1^–^6^ An analysis of NHANES data found that the relative risk of developing hypertension was increased 2.1- and 1.9-fold in obese men and women, respectively, compared with nonobese men and women.^1^

In addition to hypertension, obesity is also a risk factor for the development of numerous disorders that increase cardiovascular morbidity and mortality, including diabetes, hyperlipidemia, coronary artery disease, heart failure, and stroke.^1^,^3^ NHANES data suggest that obesity was responsible for >112,000 excess cardiovascular deaths (13% of all cardiovascular deaths) in 2004.^8^

The economic burdens of obesity and hypertension are substantial and growing. In 2008, the estimated healthcare cost attributable to obesity was $147 billion (10% of total healthcare costs) and the estimated cost attributable to hypertension was $51.0 billion in 2009. If current trends continue, these costs could reach $860 to $956 billion (16%–18% of total healthcare costs) and $200 billion, respectively, by 2030.^3^,^5^

The prevalence of obesity in patients with hypertension has increased from 25.7% during 1976 to 1980 to 50.8% during 1999 to 2004 (*P*<.05).^10^ This increase presents clinicians with many management challenges, one of which is that obesity is an independent risk factor for resistance to antihypertensive pharmacotherapy.^11^ Many obese patients will require ≥3 antihypertensive medications to control their blood pressure (BP),^11^ and as such may benefit from single-pill, triple-combination treatment.^12^

The Triple Therapy With Olmesartan Medoxomil, Amlodipine, and Hydrochlorothiazide in Hypertensive Patients Study (TRINITY; http://ClinicalTrials.gov Identifier: NCT00649389) demonstrated that the triple combination of olmesartan medoxomil (OM) 40 mg, amlodipine besylate (AML) 10 mg, and hydrochlorothiazide (HCTZ) 25 mg resulted in significantly greater reductions in both seated diastolic BP (SeDBP) and seated systolic BP (SeSBP) and enabled a significantly larger proportion of study participants to reach BP goal compared with the component dual-combination treatments.^13^ The objective of the current prespecified subgroup analysis of the TRINITY study was to evaluate the efficacy and safety of the OM 40/AML 10/HCTZ 25 mg triple-combination treatment vs the component dual-combination treatments (OM 40/AML 10 mg, OM 40/HCTZ 25 mg, and AML 10/HCTZ 25 mg) in obese (BMI ≥30 kg/m^2^) and nonobese (BMI <30 kg/m^2^) study participants at week 12, and to assess the long-term efficacy and safety of varying doses of OM/AML/HCTZ using a sequential titration algorithm tailored to the individual needs of participants, thus reflecting real-world clinical practice.

## Methods and Procedures

The details of the study design and primary results of the double-blind and open-label extension phases of the TRINTY study have been previously reported.^13^,^14^ Briefly, TRINITY was a double-blind, randomized, parallel-group study conducted at 317 clinical sites in the United States and Puerto Rico. The study consisted of a 3-week washout period, a 12-week double-blind treatment period, and a 40-week open-label extension period. It was conducted in accordance with institutional review board committee regulations and the Declaration of Helsinki.

### Study Population

Men and women 18 years and older with seated BP (SeBP) ≥140/100 mm Hg or ≥160/90 mm Hg off antihypertensive medication were eligible to participate in the study provided they did not have any history of New York Heart Association class III or IV heart failure or cerebrovascular disease; recent (≤6 months) history of myocardial infarction, percutaneous transluminal coronary revascularization, coronary artery bypass graft, or unstable angina; or presence of severe renal insufficiency (creatinine clearance <30 mL/min) or uncontrolled diabetes (glycosylated hemoglobin >9.0%). Individuals with type 1 or type 2 diabetes controlled with diet, oral hypoglycemic agents, or insulin and on a stable dose for ≥30 days were eligible to participate. All participants provided written informed consent prior to participation in any study procedures.

### Interventions

Study participants (stratified by age, race, and diabetes status) were randomized to a treatment sequence that led to their final treatment assignment (OM 40/AML 10 mg, OM 40/HCTZ 25 mg, AML 10/HCTZ 25 mg, or OM 40/AML 10/HCTZ 25 mg). Participants completing the 12-week double-blind treatment period entered a 40-week open-label extension period. All study participants were switched to triple-combination treatment OM 40/AML 5/HCTZ 12.5 mg at the beginning of the open-label extension period (week 12). Participants who did not reach BP goal (<140/90 mm Hg or <130/80 mm Hg in participants with diabetes, chronic renal disease, or chronic cardiovascular disease) within 2 weeks were randomly titrated to 1 of 2 treatments: OM 40/AML 10/HCTZ 12.5 mg or OM 40/AML 5/HCTZ 25 mg. Participants who did not reach BP goal after an additional 2 weeks (week 16) were further titrated to OM 40/AML 10/HCTZ 25 mg.

During the open-label treatment period, participants who reached BP goal remained on the same treatment; participants could be back-titrated to a lower dose of triple-combination treatment if they experienced intolerance, or be uptitrated if BP goal was not maintained. All participants were treated per investigator discretion at the conclusion of the open-label extension period (week 52).

### Study Outcomes

The primary efficacy variable in this prespecified subgroup analysis for the double-blind treatment period was the least-squares (LS) mean change in SeDBP from baseline to week 12 in the BMI subgroups (≥30 and <30 kg/m^2^). Secondary efficacy variables for this treatment period included the LS mean change in SeSBP from baseline to week 12, the proportion of study participants reaching BP goal (<140/90 mm Hg or <130/80 mm Hg in participants with diabetes, chronic kidney disease, or chronic cardiovascular disease) (subgroup prespecified analysis), the proportion of study participants (regardless of the presence of comorbidities) achieving a BP target of <140/90 mm Hg (subgroup post-hoc analysis), and the mean change from baseline in SeBP in study participants with severe hypertension (SeSBP ≥180 mm Hg or SeDBP ≥110 mm Hg) from baseline to week 12 (subgroup post-hoc analysis).

The efficacy variables for the open-label treatment period were the mean SeDBP and SeSBP at each visit week of the open-label period (data for weeks 12, 14, 16, and 52/early termination are presented in this article) and the proportion of study participants reaching SeBP goal in BMI subgroups.

Safety was assessed throughout the double-blind treatment period and the open-label extension period of the study. Safety assessments included adverse events, vital signs, physical examinations, 12-lead electrocardio-graphy, and clinical laboratory tests. Adverse events were categorized by both severity (mild, moderate, or severe) and causality (definitely, probably, possibly, unlikely, or not related to study drug), as assessed by the investigator (adverse events deemed definitely, probably, and possibly related to study drug were counted as drug-related adverse events and those deemed unlikely and not related to study drug were counted as non–drug-related adverse events).

### Statistical Analysis

The primary efficacy analysis for the 12-week double-blind treatment period included all study participants who had baseline assessment of SeDBP, received ≥1 dose of study medication, and had ≥1 post-dose SeDBP assessment. Changes in SeDBP and SeSBP within BMI subgroups were evaluated using analysis of covariance (ANCOVA) models with baseline BP as a covariate and final randomized treatment, subgroup, and final randomized treatment by subgroup interaction as fixed effects. LS mean differences and standard errors, derived from the ANCOVA model, were used to calculate mean BP change from baseline to week 12 and two-sided *P* values were used to test the significance of these changes for triple-combination treatment vs each dual-combination treatment. The proportion of study participants reaching SeBP goal and achieving SeBP target were summarized by treatment within BMI subgroups and analyzed using chi-square tests. The comparative efficacy of triple-combination treatment vs dual-combination treatment in reaching SeBP goal and target was assessed using Fisher's exact test at a 0.05 significance level. The last-observation-carried-forward (LOCF) approach was used for missing efficacy measurements during the double-blind treatment period. Differences between BMI subgroups were not assessed since the TRINITY study was not designed for these comparisons.

Efficacy in the open-label extension period was assessed in all study participants who entered the open-label period, received ≥1 dose of open-label study medication, and provided ≥1 SeBP assessment following initiation of open-label treatment. Summary statistics were used to describe SeDBP, SeSBP, and the proportion of study participants who reached SeBP goal at each open-label visit by treatment within BMI subgroups.

The primary safety population for the assessment of adverse events during the double-blind treatment period included all study participants who took ≥1 dose of study medication at or beyond the week 4 visit (ie, the first time at which study participants randomized to triple-combination treatment received this therapy). Safety in the open-label treatment period was assessed in all study participants who entered the open-label period and received ≥1 dose of open-label study medication. In both time periods, summary statistics were used to evaluate safety by treatment regimen within BMI subgroups.

## Results

### Study Population

Of the 2492 participants randomized in the TRINITY study, 1555 (62.4%) had a BMI ≥30 kg/m^2^ and 937 (37.6%) had a BMI <30 kg/m^2^. Mean weight in the BMI ≥30 kg/m^2^ subgroup was 107.2 kg and mean weight in the BMI <30 kg/m^2^ subgroup was 77.5 kg. Mean age in the BMI ≥30 kg/m^2^ subgroup was 53.9; 50.4% of study participants in the BMI ≥30 kg/m^2^ subgroup were men, 31.1% were black, 12.8% were Hispanic/Latino, and 19.0% had diabetes. Mean duration of hypertension was 10.2 years, mean baseline BP was 168.9/101.5 mm Hg, and mean prevalence of severe hypertension was 27.4% in the BMI ≥30 kg/m^2^ subgroup. Table 1 summarizes baseline demographic and clinical characteristics by randomized treatment assignment and BMI subgroup.

**Table I:** Baseline Demographic and Clinical Characteristics by Treatment and BMI Subgroup

	BMI ≥30 kg/m^2^ (n=1555)				BMI <30 kg/m^2^ (n=937)			
	OM 40/AML 10 mg (n=399)	OM 40/HCTZ 25 mg (n=399)	AML 10/HCTZ 25 mg (n=370)	OM 40/AML 10/HCTZ 25 mg (n=387)	OM 40/AML 10 mg (n=229)	OM 40/HCTZ 25 mg (n=238)	AML 10/HCTZ 25 mg (n=230)	OM 40/AML 10/HCTZ 25 mg (n=240)
Age, mean (SD), y	53.8 (10.7)	54.9 (10.8)	53.4 (10.5)	53.5 (10.6)	57.4 (11.0)	57.7 (10.6)	56.6 (11.0)	56.6 (11.9)
Men, No. (%)	194 (48.6)	197 (49.4)	206 (55.7)	186 (48.1)	131 (57.2)	142 (59.7)	128 (55.7)	134 (55.8)
Hispanic/Latino, No. (%)	46 (11.5)	50 (12.5)	49 (13.2)	54 (14.0)	44 (19.2)	35 (14.7)	49 (21.3)	42 (17.5)
White, No. (%)	276 (69.2)	261 (65.4)	244 (65.9)	255 (65.9)	156 (68.1)	161 (67.6)	151 (65.7)	160 (66.7)
Black, No. (%)	117 (29.3)	130 (32.6)	119 (32.2)	118 (30.5)	64 (27.9)	70 (29.4)	73 (31.7)	66 (27.5)
Weight, mean (SD), kg	106.6 (20.4)	107.1 (20.2)	107.9 (20.9)	107.2 (21.3)	77.2 (11.5)	77.8 (12.0)	77.0 (11.8)	78.1 (12.5)
Diabetes, No. (%)	77 (19.3)	74 (18.5)	71 (19.2)	73 (18.9)	23 (10.0)	25 (10.5)	21 (9.1)	23 (9.6)
Chronic kidney disease, No. (%)	9 (2.3)	2 (0.5)	7 (1.9)	6 (1.6)	20 (8.7)	23 (9.7)	22 (9.6)	14 (5.8)
Chronic cardiovascular disease, No. (%)	32 (8.0)	40 (10.0)	35 (9.5)	33 (8.5)	24 (10.5)	21 (8.8)	20 (8.7)	22 (9.2)
Hypertension duration, mean (SD), y	10.6 (9.8)	10.8 (9.8)	9.6 (8.8)	9.7 (9.9)	9.4 (9.9)	9.6 (9.8)	10.0 (9.3)	9.2 (8.9)
Baseline BP, mean (SD), mm Hg	168.8/101.4 (13.8/7.7)	169.1/101.2 (15.7/8.4)	169.4/101.8 (14.6/7.9)	168.3/101.6 (13.9/8.0)	167.0/100.1 (12.9/7.9)	168.7/99.7 (13.6/7.8)	168.1/100.4 (14.2/7.0)	167.1/99.9 (12.6/6.4)
Severe hypertension, No. (%)^a^	110 (27.6)	105 (26.3)	117 (31.6)	94 (24.3)	50 (21.8)	57 (23.9)	54 (23.5)	45 (18.8)

a Abbreviations: AML, amlodipine; BMI, body mass index, BP, blood pressure; HCTZ, hydrochlorothiazide; OM, olmesartan medoxomil; SD, standard deviation. ^a^Severe hypertension was defined as seated systolic BP ≥180 mm Hg or seated diastolic BP ≥110 mm Hg at baseline.

### Efficacy

**Figure 1:**
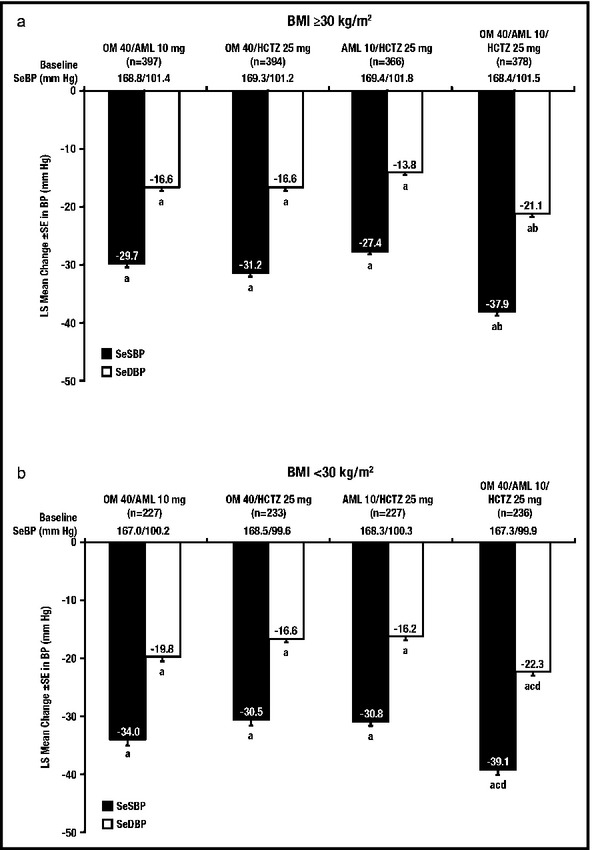
Least-squares (LS) mean reduction in seated diastolic blood pressure (SeDBP; primary efficacy variable) and seated systolic blood pressure (SeSBP) at week 12 (last observation carried forward) in study participants with body mass index (BMI) ≥30 kg/m^2^ (a) and <30 kg/m^2^ (b). Error bars represent standard error (SE). ^a^*P*<.0001 vs baseline. ^b^*P*<.0001 vs each dual-combination treatment. ^c^*P*<.005 vs OM 40/AML 10 mg. ^d^*P*<.0001 vs OM 40/HCTZ 25 mg and AML 10/HCTZ 25 mg. AML indicates amlodipine besylate; HCTZ, hydrochlorothiazide; OM, olmesartan medoxomil; SeBP, seated blood pressure.

All 4 treatment regimens resulted in significant SeDBP and SeSBP mean reductions at week 12 (LOCF) in both BMI subgroups (Figure 1). However, triple-combination treatment with OM 40/AML 10/HCTZ 25 mg resulted in significantly greater SeBP reductions compared with the component dual-combination treatments, irrespective of BMI (Figure 1). Compared with dual-combination treatments, triple-combination treatment resulted in a 6.7 to 10.5/4.5 to 7.3 mm Hg greater LS mean reduction in SeBP in study participants with a BMI ≥30 kg/m^2^ (*P*<.0001 vs each dual-combination treatment) and a 5.1 to 8.6/2.5 to 6.0 mm Hg greater LS mean reduction in SeBP in study participants with a BMI <30 kg/m^2^ (*P*<.005 vs each dual-combination treatment).

**Figure 2:**
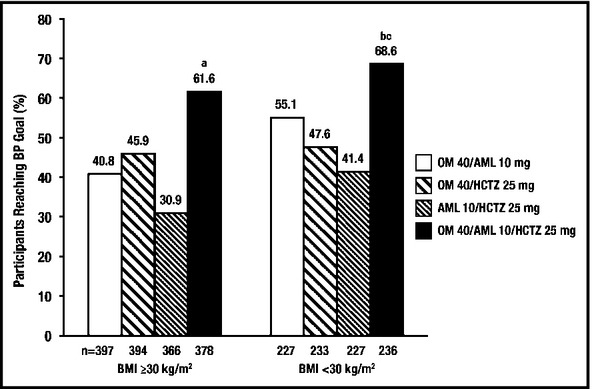
Proportion of study participants reaching blood pressure (BP) goal at week 12 (last observation carried forward). BP goal was defined as <140/90 mm Hg or <130/80 mm Hg in participants with diabetes, chronic renal disease, or chronic cardiovascular disease. ^a^*P*<.0001 vs each dual-combination treatment. ^b^*P*<.005 vs OM 40/AML 10 mg. ^c^*P*<.0001 vs OM 40/HCTZ 25 mg and AML 10/HCTZ 25 mg. AML indicates amlodipine besylate; HCTZ, hydrochlorothiazide; OM, olmesartan medoxomil.

**Figure 3:**
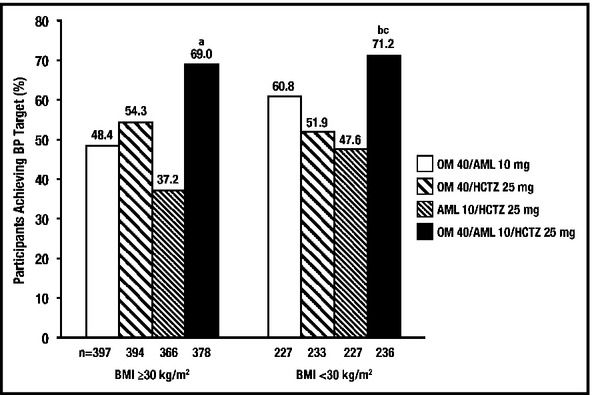
Proportion of study participants achieving blood pressure (BP) target (<140/90 mm Hg) at week 12 (last observation carried forward). ^a^*P*<.0001 vs each dual-combination treatment. ^b^*P*<.05 vs OM 40/AML 10 mg. ^c^*P*<.0001 vs OM 40/HCTZ 25 mg and AML 10/HCTZ 25 mg. AML indicates amlodipine besylate; HCTZ, hydrochlorothiazide; OM, olmesartan medoxomil.

Triple-combination treatment resulted in a significantly greater proportion of participants reaching SeBP goal (<140/90 mm Hg or <130/80 mm Hg for participants with diabetes, chronic kidney disease, or chronic cardiovascular disease) in both BMI subgroups (BMI ≥30 kg/m^2^ [*P*<.0001] and BMI <30 kg/m^2^ [*P*<.005]) (Figure 2). Overall, 61.6% and 68.6% of participants with BMI ≥30 kg/m^2^ and BMI <30 kg/m^2^, respectively, reached BP goal at week 12 on triple-combination treatment compared with each dual-combination treatment (30.9%–45.9% and 41.4%–55.1%, respectively, for the same BMI subgroups). Similarly, for both BMI subgroups, triple-combination treatment resulted in a greater proportion of study participants achieving the BP target of <140/90 mm Hg compared with each dual-combination treatment at week 12. Overall, 69.0% and 71.2% of participants with BMI ≥30 kg/m^2^ and BMI <30 kg/m^2^, respectively, achieved BP target at week 12 on triple-combination treatment (Figure 3).

**Figure 4:**
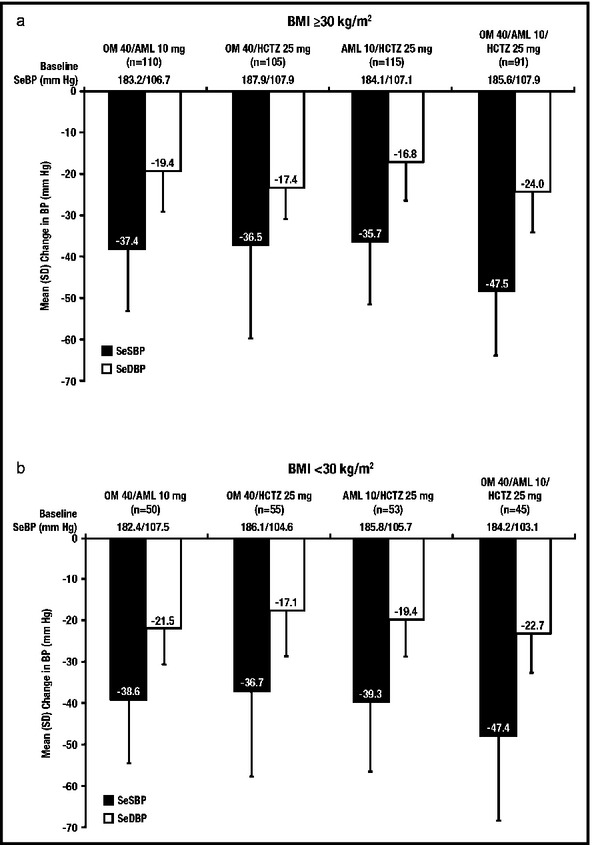
Mean reductions in seated diastolic blood pressure (SeDBP) and seated systolic blood pressure (SeSBP) at week 12 (last observation carried forward) in study participants with body mass index (BMI) ≥30 kg/m^2^ (a) and <30 kg/m^2^ (b) who had severe hypertension (SeSBP ≥180 mm Hg or SeDBP ≥110 mm Hg) at baseline (post hoc analysis). Error bars represent standard deviation (SD). AML indicates amlodipine besylate; HCTZ, hydrochlorothiazide; OM, olmesartan medoxomil; SeBP, seated blood pressure.

Likewise, triple-combination treatment resulted in greater mean reductions in SeSBP and SeDBP than the component dual-combination treatments in study participants with severe hypertension at week 12 (LOCF) in both BMI subgroups (Figure 4; statistical comparisons not performed). As a result, 40.7% of study participants with severe hypertension and BMI ≥30 kg/m^2^ and 48.9% of study participants with severe hypertension and BMI <30 kg/m^2^ who were receiving triple-combination treatment reached SeBP goal compared with 14.8% to 27.3% and 20.8% to 26.0% of study participants with BMI ≥30 kg/m^2^ and <30 kg/m^2^, respectively, who were receiving dual-combination treatments (data not shown).

**Figure 5:**
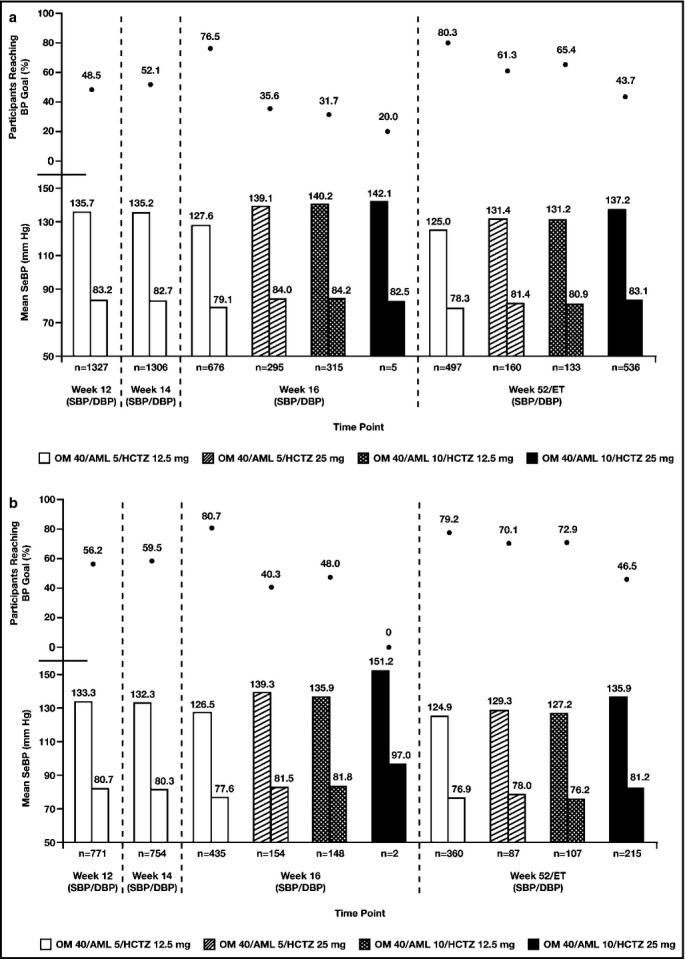
Mean seated blood pressure (SeBP) and proportion of participants reaching BP goal during the open-label extension period in obese (a) and nonobese (b) subgroups. Week 12 data are from the end of the double-blind treatment period before all participants were switched to OM 40/AML 5/HCTZ 12.5 mg. AML indicates amlodipine; DBP, diastolic blood pressure; ET, early termination; HCTZ, hydrochlorothiazide; OM, olmesartan medoxomil; SBP, systolic blood pressure.

In both BMI subgroups, the efficacy of triple-combination treatment in reducing SeBP and reaching SeBP goal was maintained or improved throughout the open-label treatment period (Figure 5). In study participants with a BMI ≥30 kg/m^2^ and <30 kg/m^2^, mean SeBP was 136/83 mm Hg and 133/81 mm Hg, respectively, at the start of the open-label period and 131/81 mm Hg and 129/78 mm Hg, respectively, at week 52/early termination. Similarly, the proportions of study participants with BMI ≥30 kg/m^2^ and <30 kg/m^2^ reaching SeBP goal were 49% and 56%, respectively, at the start of the open-label period and 63% and 67% at week 52/early termination.

### Safety

No new safety concerns were identified during the double-blind treatment period that were not already known to occur with the individual component therapies. Of the 1449 study participants with a BMI ≥30 kg/m^2^, 840 (58.0%) had a treatment-emergent adverse event (TEAE), 365 (25.2%) had a drug-related TEAE, 29 (2.0%) had a serious adverse event, and 20 (1.4%) discontinued study participation because of a drug-related TEAE. Similarly, of the 853 study participants with a BMI <30 kg/m^2^, 447 (52.4%) had a TEAE, 220 (25.8%) had a drug-related TEAE, 6 (0.7%) had a serious adverse event, and 12 (1.4%) discontinued study participation because of a drug-related TEAE. Table 2 summarizes the prevalence and type of TEAEs within BMI subgroups by treatment group. Across BMI and treatment groups, most TEAEs and drug-related TEAEs were considered mild or moderate in severity. The most common TEAEs (≥5%) that occurred in study participants with BMI ≥30 kg/m^2^ were peripheral edema (6.7%), dizziness (6.2%), headache (6.1%), and fatigue (5.2%), and the most common TEAEs (≥5%) occurring in study participants with BMI <30 kg/m^2^ were dizziness (8.3%), headache (7.2%), and fatigue (5.9%).

**Table II:** Study Participants With TEAEs at Week 12

	BMI ≥30 kg/m^2^ (n=1449)				BMI <30 kg/m^2^ (n=853)			
	OM 40/AML 10 mg (n=380)	OM 40/HCTZ 25 mg (n=371)	AML 10/HCTZ 25 mg (n=339)	OM 40/AML 10/HCTZ 25 mg (n=359)	OM 40/AML 10 mg (n=216)	OM 40/HCTZ 25 mg (n=209)	AML 10/HCTZ 25 mg (n=213)	OM 40/AML 10/HCTZ 25 mg (n=215)
All TEAEs^a^	209 (55.0)	207 (55.8)	202 (59.6)	222 (61.8)	99 (45.8)	112 (53.6)	123 (57.7)	113 (52.6)
Severe TEAEs	20 (5.3)	10 (2.7)	9 (2.7)	14 (3.9)	4 (1.9)	7 (3.3)	9 (4.2)	10 (4.7)
Drug-related TEAEs^b^	89 (23.4)	79 (21.3)	92 (27.1)	105 (29.2)	49 (22.7)	42 (20.1)	72 (33.8)	57 (26.5)
Severe drug-related TEAEs	5 (1.3)	2 (0.5)	1 (0.3)	4 (1.1)	4 (1.9)	2 (1.0)	1 (0.5)	3 (1.4)
Discontinuations								
TEAEs	5 (1.3)	6 (1.6)	8 (2.4)	11 (3.1)	1 (0.5)	6 (2.9)	3 (1.4)	12 (5.6)
Drug-related TEAEs^b^	3 (0.8)	3 (0.8)	3 (0.9)	11 (3.1)	1 (0.5)	2 (1.0)	2 (0.9)	7 (3.3)
TEAEs (≥3% in any treatment group)								
Dizziness	17 (4.5)	37 (10.0)	5 (1.5)	31 (8.6)	12 (5.6)	21 (10.0)	12 (5.6)	26 (12.1)
Headache	22 (5.8)	26 (7.0)	17 (5.0)	24 (6.7)	20 (9.3)	12 (5.7)	16 (7.5)	13 (6.0)
Fatigue	21 (5.5)	22 (5.9)	18 (5.3)	14 (3.9)	13 (6.0)	9 (4.3)	18 (8.5)	10 (4.7)
Edema, peripheral	30 (7.9)	5 (1.3)	27 (8.0)	35 (9.7)	12 (5.6)	1 (0.5)	19 (8.9)	9 (4.2)
Upper respiratory tract infection	17 (4.5)	12 (3.2)	11 (3.2)	11 (3.1)	9 (4.2)	6 (2.9)	3 (1.4)	5 (2.3)
Urinary tract infection	7 (1.8)	6 (1.6)	5 (1.5)	11 (3.1)	1 (0.5)	0	2 (0.9)	3 (1.4)
Nasopharyngitis	6 (1.6)	13 (3.5)	7 (2.1)	16 (4.5)	5 (2.3)	7 (3.3)	9 (4.2)	4 (1.9)
Nausea	4 (1.1)	13 (3.5)	7 (2.1)	8 (2.2)	8 (3.7)	9 (4.3)	5 (2.3)	9 (4.2)
Diarrhea	6 (1.6)	9 (2.4)	5 (1.5)	11 (3.1)	8 (3.7)	3 (1.4)	4 (1.9)	4 (1.9)
Joint swelling	10 (2.6)	1 (0.3)	6 (1.8)	5 (1.4)	7 (3.2)	1 (0.5)	10 (4.7)	7 (3.3)
Muscle spasms	11 (2.9)	6 (1.6)	8 (2.4)	13 (3.6)	1 (0.5)	8 (3.8)	5 (2.3)	5 (2.3)
Blood potassium decreased	1 (0.3)	1 (0.3)	7 (2.1)	2 (0.6)	0	2 (1.0)	7 (3.3)	3 (1.4)
Hypokalemia	2 (0.5)	3 (0.8)	13 (3.8)	4 (1.1)	0	0	12 (5.6)	0

Abbreviations: AML, amlodipine besylate; BMI, body mass index; HCTZ, hydrochlorothiazide; OM, olmesartan medoxomil; TEAE, treatment-emergent adverse event. Data are presented as numbers (percentages).

a TEAEs were adverse events that emerged during treatment (absent pretreatment or worsened relative to pretreatment). TEAEs are defined as having a start date on/after the first dose of double-blind study medication and up to the first dose of open-label study medication for participants continuing into the open-label period, or, for early terminated participants, up to and including 14 days after the last dose date of double-blind study medication. All TEAEs are counted under the treatment the participant received from week 4 to week 12.

b Drug-related was defined as definitely, probably, or possibly related to randomized study medication.

During the open-label treatment period, 984 of 1332 (73.9%) study participants with a BMI ≥30 kg/m^2^ and 531 of 780 (68.1%) study participants with a BMI <30 kg/m^2^ had an adverse event (Table 3). Similar to the double-blind treatment period, most of these adverse events were considered to be mild or moderate in severity and unrelated to study medication (Table 3). Adverse events that occurred in ≥5% of study participants during this period were dizziness (7.0%), upper respiratory tract infection (6.9%), peripheral edema (6.8%), nasopharyngitis (5.3%), and urinary tract infection (5.2%) in participants with BMI ≥30 kg/m^2^ and dizziness (9.1%) and nasopharyngitis (5.5%) in participants with BMI <30 kg/m^2^ (Table 3).

**Table III:** Study Participants With AEs During the Open-Label Treatment Period by Onset Dosing Regimen

	BMI ≥30 kg/m^2^ (n=1332)				BMI <30 kg/m^2^ (n=780)			
	OM 40/AML 5/HCTZ 12.5 mg (n=1332)	OM 40/AML 5/HCTZ 25 mg (n=426)	OM 40/AML 10/HCTZ 12.5 mg (n=430)	OM 40/AML 10/HCTZ 25 mg (n=560)	OM 40/AML 5/HCTZ 12.5 mg (n=780)	OM 40/AML 5/HCTZ 25 mg (n=201)	OM 40/AML 10/HCTZ 12.5 mg (n=222)	OM 40/AML 10/HCTZ 25 mg (n=230)
All AEs^a^	640 (48.0)	145 (34.0)	158 (36.7)	339 (60.5)	349 (44.7)	83 (41.3)	86 (38.7)	128 (55.7)
Severe AEs	49 (3.7)	13 (3.1)	16 (3.7)	32 (5.7)	29 (3.7)	8 (4.0)	9 (4.1)	15 (6.5)
Drug-related AEs^b^	192 (14.4)	48 (11.3)	43 (10.0)	112 (20.0)	119 (15.3)	25 (12.4)	26 (11.7)	44 (19.1)
Discontinuations								
AEs	30 (2.3)	7 (1.6)	8 (1.9)	19 (3.4)	44 (5.6)	3 (1.5)	4 (1.8)	12 (5.2)
AEs starting in open-label extension period	27 (2.0)	7 (1.6)	8 (1.9)	17 (3.0)	40 (5.1)	3 (1.5)	3 (1.4)	11 (4.8)
Drug-related AEs^b^	15 (1.1)	5 (1.2)	4 (0.9)	6 (1.1)	30 (3.8)	2 (1.0)	3 (1.4)	7 (3.0)
AEs (≥3% in any treatment group)								
Dizziness	53 (4.0)	12 (2.8)	10 (2.3)	23 (4.1)	38 (4.9)	10 (5.0)	12 (5.4)	15 (6.5)
Headache	31 (2.3)	8 (1.9)	9 (2.1)	18 (3.2)	16 (2.1)	8 (4.0)	8 (3.6)	8 (3.5)
Edema, peripheral	32 (2.4)	4 (0.9)	18 (4.2)	42 (7.5)	10 (1.3)	4 (2.0)	8 (3.6)	6 (2.6)
Upper respiratory tract infection	50 (3.8)	7 (1.6)	12 (2.8)	25 (4.5)	22 (2.8)	4 (2.0)	4 (1.8)	8 (3.5)
Urinary tract infection	44 (3.3)	9 (2.1)	5 (1.2)	18 (3.2)	15 (1.9)	6 (3.0)	2 (0.9)	5 (2.2)
Nasopharyngitis	33 (2.5)	9 (2.1)	8 (1.9)	22 (3.9)	22 (2.8)	6 (3.0)	6 (2.7)	9 (3.9)
Back pain	20 (1.5)	5 (1.2)	7 (1.6)	11 (2.0)	11 (1.4)	3 (1.5)	8 (3.6)	5 (2.2)
Cough	32 (2.4)	7 (1.6)	5 (1.2)	16 (2.9)	12 (1.5)	9 (4.5)	3 (1.4)	2 (0.9)
Arthralgia	20 (1.5)	6 (1.4)	6 (1.4)	17 (3.0)	7 (0.9)	1 (0.5)	2 (0.9)	10 (4.3)

Abbreviations: AEs, adverse events; AML, amlodipine besylate; BMI, body mass index, HCTZ, hydrochlorothiazide; OM, olmesartan medoxomil. Data are presented as numbers (percentages).

a Adverse events starting before the open-label extension period and not resolved by week 12 were counted under the final dosing regimen.

b Drug-related was defined as definitely, probably, or possibly related to randomized study medication.

No important differences were noted between BMI subgroups in mean (standard deviation) exposure to double-blind study medication (BMI ≥30 kg/m^2^, 83 [10] days; BMI <30 kg/m^2^, 83 [10] days) or open-label study medication (BMI ≥30 kg/m^2^, 259 [66] days; BMI <30 kg/m^2^, 252 [75] days). In addition, there did not appear to be a clinically relevant difference in the overall incidence of adverse events between study participants with BMI ≥30 kg/m^2^ and study participants with BMI <30 kg/m^2^ during either the 12-week double-blind treatment period or 40-week open-label treatment period (Tables 2 and 3).

## Discussion

This prespecified subgroup analysis of a large, multicenter, randomized, parallel-group study demonstrated the efficacy and safety of triple-combination treatment with OM 40/AML 10/HCTZ 25 mg in obese participants with hypertension. Triple-combination treatment resulted in greater mean reductions in SeBP and enabled larger proportions of study participants to achieve BP goal and target compared with the component, dual-combination treatments during the double-blind treatment period in both BMI subgroups. In addition, a treatment algorithm employing varying doses of the triple-combination agents that was modeled after real-world clinical practice for titrating patients to goal resulted in long-term BP reductions during the open-label treatment period that were comparable with those obtained during the double-blind treatment period in both BMI subgroups, demonstrating the maintenance of antihypertensive efficacy of the triple-combination treatment. Triple-combination treatment was well tolerated during both treatment periods in both BMI subgroups.

Obesity is a global epidemic that affects all age, sex, and race/ethnic groups.^3^ NHANES data indicate that approximately 16.9% of children 2 to 19 years of age and 34.6% of adults in the United States are currently obese.^3^ Moreover, the prevalence of obesity was 28.1% (men) and 34.0% (women) for 1999 to 2002 and increased to 34.4% and 36.1% in 2007 to 2010, respectively. In US children 6 to 11 years of age, the prevalence of obesity was 17.0% in 1999 to 2002 and increased to 18.8% in 2007 to 2010.^3^ These increases are caused, at least in part, by population factors, including high-caloric diet and sedentary lifestyle.^1^–^3^ Data from the 2010 National Health Interview Survey (NHIS) indicate that approximately one third of US adults do not engage in leisure-time activities involving at least light-intensity physical activity for ≥10 minutes.^3^

Data from the Framingham Heart Study clearly demonstrate that obesity increases cardiovascular risk.^1^ An early evaluation of 26-year follow-up data found that obesity was a significant and independent risk factor for cardiovascular diseases, including coronary heart disease, coronary death, and congestive heart failure in both men and women, even after controlling for major cardiovascular risk factors.^15^ Likewise, a subsequent evaluation of 44-year follow-up data found that, compared with individuals with a BMI of 18.5 to 24.9 kg/m^2^, individuals with a BMI ≥30 kg/m^2^ had significant increases in the relative risks of hypertension (men, 2.2; women, 2.6), diabetes (men, 1.9; women, 1.4), angina (men, 1.8; women, 1.6), total coronary heart disease (men, 1.6; women, 1.5), and total cardiovascular disease (men, 1.4; women, 1.4).^16^

Obesity appears to be an important risk factor for the development of primary hypertension.^2^ In an analysis of NHANES data from 2007 to 2008, individuals with hypertension were 3.9 times more likely to have a BMI ≥25 kg/m^2^ (79.4%) and 2.3 times more likely to have a BMI ≥30 kg/m^2^ (46.5%) than to have a BMI <25 kg/m^2^ (20.6%).^17^ Since both obesity and hypertension are major risk factors for cardiovascular disease, achieving adequate BP control is crucial in these individuals.^12^ Current guidelines recommend weight loss as an effective means of lowering BP in obese individuals with hypertension.^1^,^12^ However, most obese individuals find it difficult to achieve and/or maintain adequate weight reduction, necessitating the use of antihypertensive agents to lower BP.

Current hypertension guidelines do not provide specific recommendations for the pharmacologic management of obese individuals with hypertension. General recommendations include initiating antihypertensive therapy with two agents if systolic BP is >20 mm Hg or diastolic BP is >10 mm Hg above goal and using long-acting agents that provide 24-hour efficacy with once-daily administration to keep the therapeutic regimen simple and improve adherence.^12^–^18^ Since obese individuals with hypertension frequently have other cardiovascular risk factors that require pharmacotherapy, such as hyperlipidemia and diabetes, maintaining a simple therapeutic regimen may be especially important in these individuals.^3^

Early and effective BP control is important since it can prevent or delay long-term complications associated with hypertension.^19^–^20^ Hypertension in obese persons is caused by a combination of factors, including volume expansion, increased cardiac output, enhanced neurohormonal activation, hyperinsulinemia, renal dysfunction, and obstructive sleep apnea.^2^–^21^ As a result, interfering with a single BP mechanism is unlikely to achieve adequate control.^21^ Available data suggest that approximately 75% of individuals with hypertension will require combination therapy and 25% may need 3 antihypertensive agents to reach BP goal.^21^–^25^ These percentages may be even higher in obese individuals with hypertension.^11^–^29^ In this subgroup analysis, 536 participants (40%) with BMI ≥30 kg/m^2^ were receiving the highest triple-combination dose regimen (OM 40/AML 10/HCTZ 25 mg) at week 52 compared with 215 participants (28%) with BMI <30 kg/m^2^, providing evidence that patients with higher BMI are difficult to treat and often require higher doses of therapy.

NCHS data indicate that cardiovascular disease is responsible for 69% of the excess mortality associated with obesity.^8^ Since registry data indicate that only 34% of obese individuals with comorbid diabetes and/or cardiovascular disease currently attain guideline-recommended BP targets,^26^ treatment of hypertension in individuals with obesity may need to be more aggressive. Single-pill, triple-combination treatment may provide benefit in terms of increased adherence through reduction in pill burden and simplification of the therapeutic regimen.

## Conclusions

Triple-combination treatment with OM 40/AML 10/HCTZ 25 mg was as safe as and more effective than the component dual-combination treatments in difficult-to-treat obese participants with hypertension. In both BMI subgroups, triple-combination treatment resulted in significantly greater mean reductions in SeSBP and SeDBP, enabling a significantly larger proportion of study participants to reach BP goal and target compared with the component dual-combination treatments during the double-blind treatment period. Triple-combination treatment was also well tolerated and safely maintained BP reductions during the open-label treatment period. As a result, triple-combination treatment with OM 40/AML 10/HCTZ 25 mg may provide a safe and effective therapeutic option for difficult-to-treat obese individuals with hypertension whose BP is not adequately controlled with dual-combination regimens.
